# A case of microsatellite instability-high clinically advanced castration-resistant prostate cancer showing a remarkable response to pembrolizumab sustained over at least 18 months

**DOI:** 10.1101/mcs.a006194

**Published:** 2022-06

**Authors:** Kosuke Shimizu, Takeshi Sano, Kei Mizuno, Takuro Sunada, Noriyuki Makita, Hiroki Hagimoto, Takayuki Goto, Atsuro Sawada, Masakazu Fujimoto, Kentaro Ichioka, Osamu Ogawa, Takashi Kobayashi, Shusuke Akamatsu

**Affiliations:** 1 Department of Urology, Kyoto University Hospital, Kyoto, 606-8507, Japan;; 2 Department of Diagnostic Pathology, Kyoto University Hospital, Kyoto, 606-8507, Japan;; 3 Ichioka Urological Clinic, Kyoto, 604-0837, Japan

**Keywords:** macroscopic hematuria, prostate cancer, rectal fistula, urinary retention

## Abstract

Defective DNA mismatch repair genes can lead to microsatellite instability (MSI)-high status in prostate cancer (PC). Accumulation of replication errors in DNA leads to the production of abundant neoantigens, which could be targets for immune checkpoint inhibitors (CPIs). However, the incidence of MSI-high PC is low, and not all patients show a satisfactory therapeutic response to CPIs. Here, we present the case of a patient with MSI-high castration-resistant PC who showed a remarkable and durable response to pembrolizumab. The patient was resistant to abiraterone, docetaxel, and cabazitaxel and was suffering from multiple tumor-associated or treatment-related complications, such as urinary tract infection, infective endocarditis, and uncontrollable prostatic hemorrhage. Soon after the start of pembrolizumab therapy, the patient showed a dramatic decrease in prostate-specific antigen from 35.67 ng/mL to an undetectable level and a remarkable reduction in the size of a massive prostate mass and lymph node metastases, with an absence of treatment-related complications. Specimens from the transurethral resection of prostate cancer during cabazitaxel treatment for control of prostate bleeding and also that from the prostate biopsy at initial diagnosis revealed MSI-high status. Immunohistochemistry showed loss of MSH2 and MSH6, and whole-exome sequencing revealed an approximate tumor mutation burden of 61 mutations/Mb as well as biallelic loss of *MSH2*. Pembrolizumab could show a significant effect even in a heavily treated patient with MSI-high advanced PC. Accumulation of detailed clinical and genomic information of cases of MSI-high PC treated with pembrolizumab is necessary for optimal patient selection.

## INTRODUCTION

There are various treatments for castration-resistant prostate cancer (CRPC) approved by the Ministry of Health, Labor and Welfare in Japan, including androgen receptor-axis-targeted (ARAT) agents, radium-223, docetaxel, and cabazitaxel (Tannock et al. 2004; de Bono et al. 2010; Parker et al. 2013; Ryan et al. 2013; Beer et al. 2014). However, these treatments for CRPC prolong overall survival (OS) by only a few months, and the tumors eventually gain treatment resistance (Komura et al. 2018; Teo et al. 2019). Pembrolizumab, an immune checkpoint inhibitor (CPI), has been reported to be effective for microsatellite instability (MSI)-high solid tumors in the KEYNOTE-016 trial and has been approved for MSI-high solid tumors including prostate cancer (PC) (Le et al. 2015). Recently, several small case series of MSI-high PC treated with pembrolizumab have been published showing that deep response can be observed in some patients (Abida et al. 2019; Barata et al. 2020; Graham et al. 2020; Marabelle et al. 2020). In addition, a few case reports on MSI-high PC treated with pembrolizumab have been published with sufficient descriptions of the patient's clinical course (Costa et al. 2019; Fujiwara et al. 2020; Han et al. 2020; Ravindranathan et al. 2021). Nonetheless, clinical and molecular characteristics of patients with MSI-high PC who respond to pembrolizumab are not well-defined, and detailed information on the clinical course of MSI-high PC patients treated with pembrolizumab remains sparse. Herein, we report the case of a patient with MSI-high CRPC refractory to abiraterone, docetaxel, and cabazitaxel, who was treated with pembrolizumab and showed a remarkable and durable response.

## RESULTS

### Clinical Presentation and Genomic Analysis of Tumor Tissue

**Figure 1.**
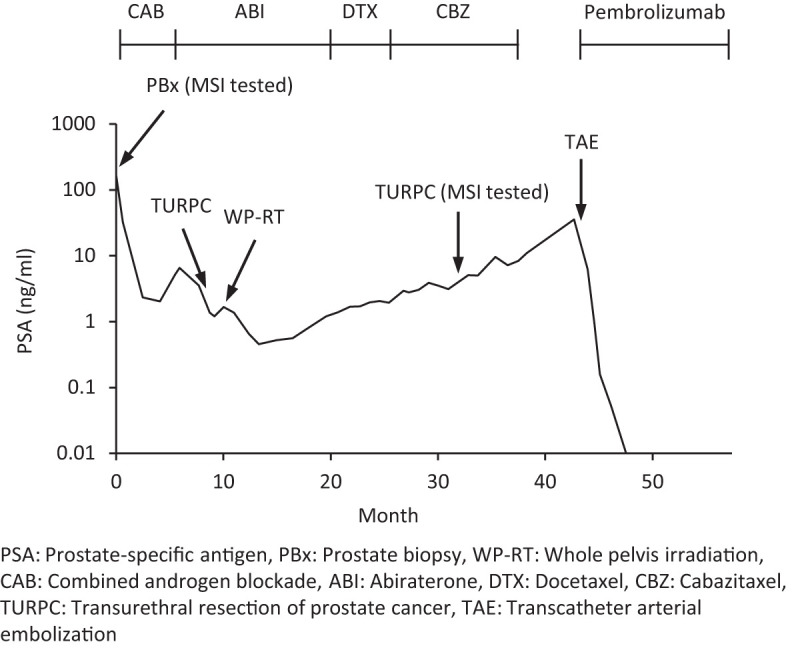
Prostate-specific antigen values along the patient's clinical course.

A 67-yr-old man visited a local urology clinic with a chief complaint of macroscopic hematuria and was diagnosed with PC by transperineal prostate biopsy (PBx). The patient was referred to our hospital for treatment of PC in April 2017. He had no past illness or family history of malignancies. Computed tomography (CT) detected enlargement of a left obturator lymph node, and bone scintigraphy was negative. Prostate-specific antigen (PSA) levels along the patient's clinical course are summarized in Figure 1. Combined androgen blockade (CAB) with leuprolide acetate and bicalutamide was initiated for a Gleason score (GS) of 5 + 4, cT4N1M0 PC. Although the PSA value decreased from 160 ng/mL to 2.04 ng/mL, it started increasing only 4 mo after the initiation of CAB. The patient was diagnosed with CRPC, and abiraterone was started 6 mo after the initial diagnosis.

Nine months after the diagnosis, transurethral resection of PC (TURPC) was performed to alleviate urinary symptoms caused by the local progression of the tumor. The pathological diagnosis was acinar adenocarcinoma mixed with small-cell carcinoma. The majority of the tumor was acinar adenocarcinoma–positive for PSA; however, ∼10% of the tumor showed small-cell morphology, which stained positive for chromogranin A and synaptophysin. One month later, whole pelvis irradiation of 72 Gy targeting the prostate and lymph node metastases was given. However, the enlargement of the right internal iliac lymph node became detectable, and the PSA value increased again, with massive local progression of the local tumor necessitating a suprapubic cystostomy. Neither six cycles of docetaxel nor 13 cycles of cabazitaxel were effective. During cabazitaxel therapy, the patient underwent a second TURPC to manage the hemorrhage from the PC, which showed adenocarcinoma and no residual small-cell carcinoma. Urinary diversion via a transverse colon conduit was necessary 35 mo after initial diagnosis because of frequent urinary tract infection (UTI) and uncontrollable bleeding from the prostate. Although the UTI appeared to be well-controlled, infective endocarditis developed from the tumor-derived exudate pooling in the empty bladder. Thus, a transurethral catheter was placed to remove fluid collection in the bladder, and aortic and mitral valve replacement was performed 38 mo after the initial diagnosis of PC.

**Figure 2.**
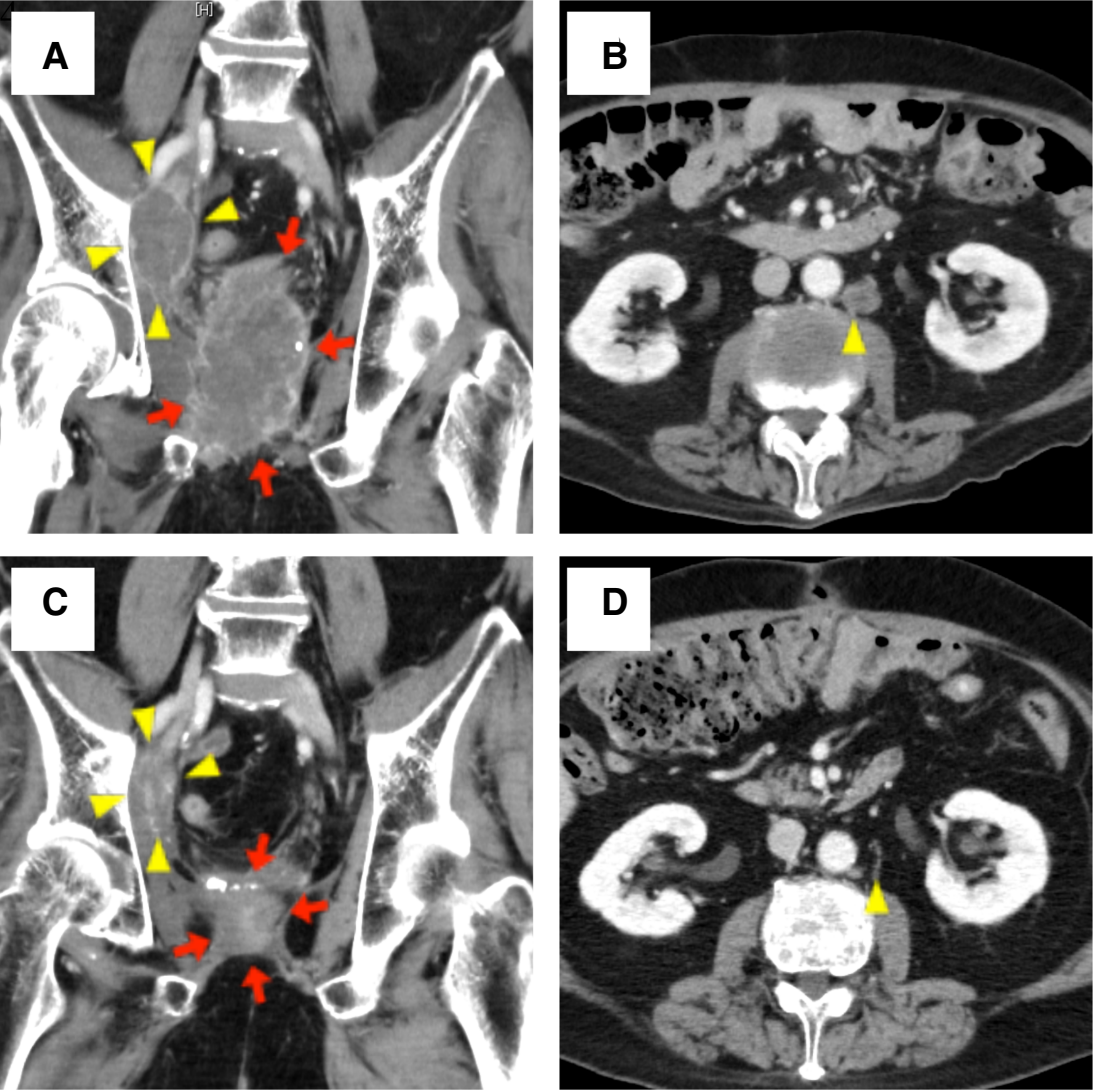
Images of abdominal contrast–enhanced computed tomography before (*A*,*B*), and 10 mo after (*C*,*D*) the start of pembrolizumab treatment. (*A*,*C*) Coronal images show a remarkable reduction in the size of the local lesion and the right obturator lymph node. (*B*,*D*) Axial images showing the disappearance of the para-aortic lymph node.

Because the patient's performance status declined after the cardiac surgery, the patient was determined to be no longer fit for cytotoxic chemotherapy. Therefore, we performed MSI testing using the pathologic specimen of the second TURPC, which was covered by the public medical insurance in Japan for the patients with solid tumors for whom standard therapy options have been exhausted. MSI-high status was confirmed, and pembrolizumab (200 mg/body, every 3 wk) was started 43 mo after the initial diagnosis. Soon after the first cycle of pembrolizumab, transcatheter arterial embolization (TAE) via the femoral artery was performed to control local bleeding from the prostate. Nineteen days after pembrolizumab administration, a rectocutaneous fistula was identified. Abdominal contrast–enhanced CT showed invasion of the PC to the rectum and a fistula between the invaded PC lesion and the surface of his left buttock. After the second cycle of pembrolizumab, a colostomy using the transverse colon was constructed to treat the fistula and prevent rectal obstruction. After the surgery, the fistula spontaneously closed, and, surprisingly, the PSA value dramatically decreased from the maximum value of 35.67 ng/mL to an undetectable level (<0.01 ng/mL) following the fourth cycle of pembrolizumab. CT scan also showed a remarkable decrease in the size of the lymph node metastases and the local PC lesion (Fig. 2). Pembrolizumab treatment has been continued every 3 wk, and PSA value remains at an undetectable level 18 mo after the start of pembrolizumab treatment. Tumor-derived exudate reduced to <10 mL/d, and the urethral catheter was removed.

**Figure 3.**
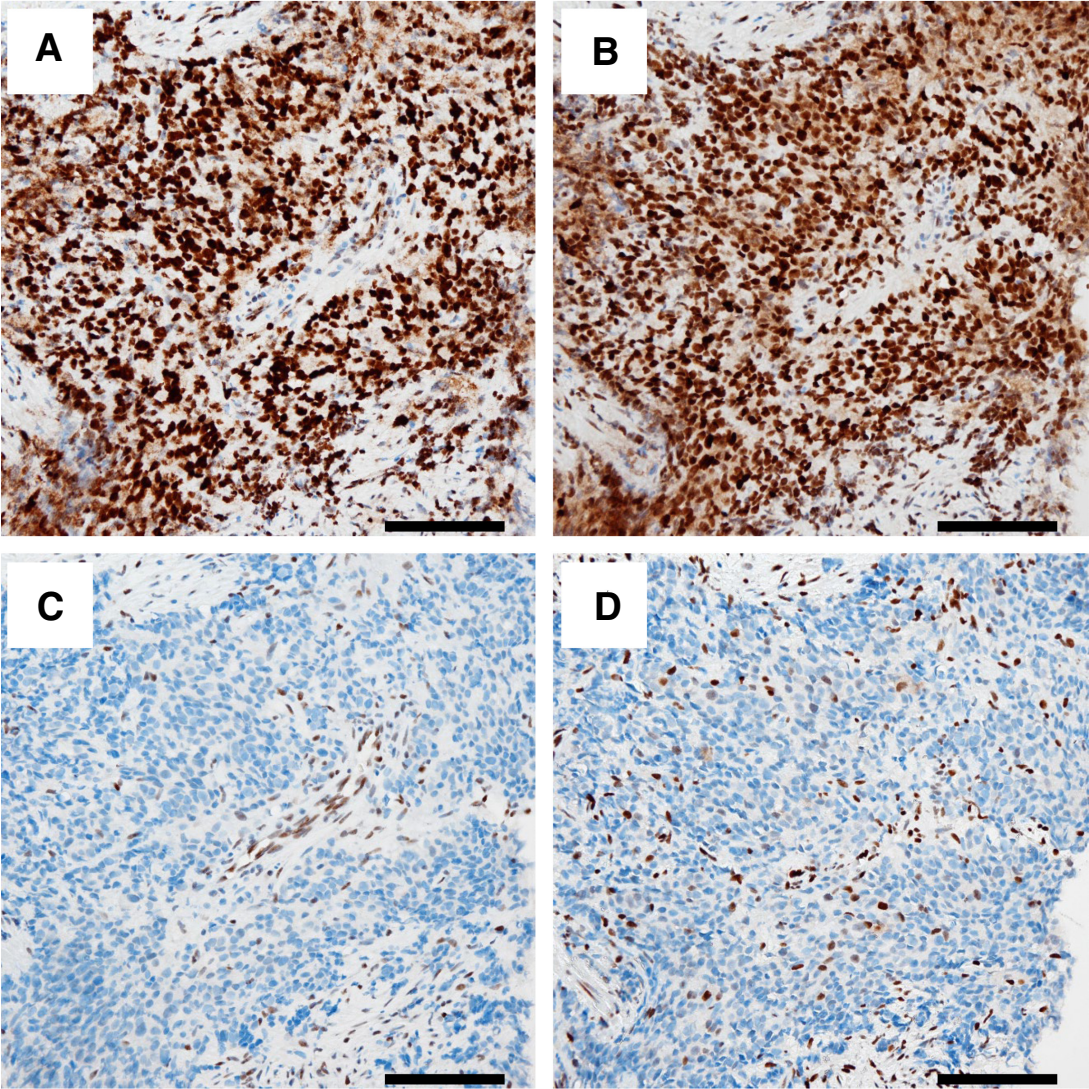
Microscopic images of immunohistochemistry using prostate biopsy specimens at 200× magnification. The tumor tissues show positive staining for PMS2 (*A*) and MLH1 (*B*) and negative staining for MSH2 (*C*) and MSH6 (*D*). Scale bar, 100 µm.

**Figure 4.**
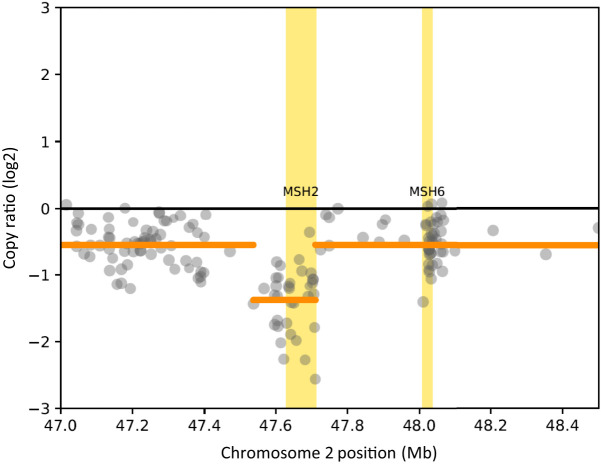
A scatter plot showing the copy-number variations of MSH2 and MSH6.

To examine if we could have diagnosed MSI-high status at an earlier time point during treatment, we also confirmed that the specimen of initial PBx was MSI-high. Immunohistochemistry revealed loss of MSH2 and MSH6 and intact PMS2 and MLH1 in both PBx (Fig. 3) and the second TURPC samples (data not shown). Whole-exome sequencing using the second TURPC sample was also performed to investigate the status of the DNA mismatch repair genes and tumor mutation burden (TMB). The number of total reads and total mapped reads for the tumor sample were 70,738,800 and 70,653,600 (99.88%), and those for the matched leukocyte sample were 96,199,800 and 96,009,200 (99.80%), respectively. The average coverage at targeted regions for tumor and leukocyte DNA were 130 and 160, respectively. The complete loss of *MSH2* was confirmed (Fig. 4). For *MSH6*, only a monoallelic loss was detected without an evident loss of a second hit (Fig. 4). Overall, there were 1820 mutations (∼61 mutations/Mb) across the whole exome, compatible with high TMB. Among the known recurrently altered genes in PC, frameshift deletions of *ARID1A, ATR, NBN, PTEN, ZMYM3*, and *SMARCA1* as well as nonsynonymous single-nucleotide variants (SNVs) of *CHD1*, *KMT2D, TP53*, and *CDK12* were found (Table 1; Supplemental Table S1).

**Table 1.** Representative gene alterations in transurethral resection of prostate cancer tissues detected using whole-exome sequencing

Gene	Chromosome	Genomic position	Reference base	Variant base	AA change	Variant type	Total number of reads	Number of variant reads (n)	VAF (%)
*ARID1A*	1	27,101,268	C	–	A1517fs	Frameshift deletion	397	115	29.0
*ATR*	3	142,274,740	T	–	I774fs	Frameshift deletion	45	14	31.1
*CHD1*	5	98,209,341	C	T	C1176Y	Nonsynonymous SNV	40	14	35.0
*CHD1*	5	98,228,323	G	A	R696C	Nonsynonymous SNV	27	7	25.9
*NBN*	8	90,967,512	T	–	R466fs	Frameshift deletion	31	10	32.3
*PTEN*	10	89,717,770	A	–	L265fs	Frameshift deletion	46	14	30.4
*KMT2D*	12	49,427,369	G	A	R3707X	Nonsense	158	79	50.0
*TP53*	17	7,577,536	T	C	R249G	Nonsynonymous SNV	103	28	27.2
*TP53*	17	7,578,389	G	A	R181C	Nonsynonymous SNV	122	48	39.3
*CDK12*	17	37,657,655	C	T	R858W	Nonsynonymous SNV	66	24	36.4
*ZMYM3*	X	70,467,749	CA	–	V661fs	Frameshift deletion	108	78	72.2
*SMARCA1*	X	128,621,084	T	–	M710fs	Frameshift deletion	74	50	67.6

(AA) Amino acid, (SNV) single-nucleotide variant, (VAF) variant allele frequency.

## DISCUSSION

Previous reports have described that the overall incidence of MSI-high status in PC patients is as low as ∼3% (Abida et al. 2019; Barata et al. 2020). Among the reported cases, approximately one-third of patients treated with pembrolizumab showed a durable decline in PSA values >99% (Abida et al. 2019; Barata et al. 2020). A previous study also reported 15 men with deficient mismatch repair (dMMR) and/or MSI-high metastatic PC who received pembrolizumab monotherapy and had sufficient clinical data to evaluate PSA response, among which seven (47%) showed a decline in PSA values >90% (Graham et al. 2020). In addition, five cases of MSI-high PC treated with pembrolizumab showing a remarkable response have been reported with sufficient data presentation (Costa et al. 2019; Fujiwara et al. 2020; Han et al. 2020; Ravindranathan et al. 2021). PSA values of three cases became <0.01 ng/mL after treatment. Among them, the case reported by Han et al. and the present case sequentially underwent local therapy (i.e., radiotherapy and TAE) during pembrolizumab treatment. These two cases showed undetectable PSA levels soon after the start of pembrolizumab treatment. This may suggest a potential synergistic effect of local therapy by releasing abundant tumor neoantigens into the blood stream to enhance the treatment effect of pembrolizumab. Further accumulation of data is warranted.

Despite these accumulations of clinical reports, there is no biological predictive marker for a good response of MSI-high PC to CPIs. Programmed death-ligand 1 (PD-L1) expression has been reported to be a biomarker for the prediction of CPI responders in advanced melanoma, non-small-cell lung cancer, and cervical cancer (Topalian et al. 2016). However, the association between PD-L1 expression and response to CPIs in patients with CRPC has been controversial (Hansen et al. 2018; Antonarakis et al. 2020; de Almeida et al. 2020). The tumor tissue did not show programmed cell death protein 1 expression in the present case (data not shown). In the present study, we also performed whole-exome sequencing of the tumor tissue to explore if any other gene alterations that are potentially relevant to the significant treatment effect could be identified. *ATR* is the master regulator of single-strand DNA damage repair, and *TP53* is known to be a strong prognostic factor in CRPC. Whether alterations in these two genes had any clinical relevance in the present case remains unknown. Wu et al. reported that biallelic *CDK12* alterations cause tandem duplications, leading to genomic instability and production of many fusion genes that could also result in abundant neoantigens and response to CPIs, irrespective of dMMR status (Wu et al. 2018). Although many of the pathogenic alterations of *CDK12* reported to be pathogenic in clinical sequencing are allelic loss or truncating SNVs, R858 is one of the two canonical CDK arginines and potentially a hotspot for mutations in *CKD12* associated with functionality of this gene. R858 arginine and R882 arginine has been reported to form ionic interactions with the phosphate group for full activation of the gene (Bösken et al. 2014). Importantly, of the 35 cases with biallelic *CDK12* aberrations reported, two harbored the R858W variant observed in the present study (Wu et al. 2018). However, in the present case, the remaining *CDK12* allele was intact and no evidence of tandem duplication was detected in the available exome data. In any case, because one allele of *CDK12* was intact in the tumor, it appears unlikely that *CDK12* alteration alone was important to the response to pembrolizumab, but additional study of clinical response to CPI in the setting of inactivation of one allele in *CDK12* and the presence of dMMR is warranted. TMB in metastatic PC is generally much <10 mutations/Mb (Markert et al. 2011). In contrast, TMB among MSI-high PC cases varies. In the present case, the presence of MSI-high status at diagnosis and an increase of TMB over time may have affected the clinical outcome. Future studies are needed to evaluate if TMB values may provide additional prediction accuracy for CPI response in MSI-high PC.

In the present case, even though the patient was heavily pretreated and the patient's performance status was very poor, surprisingly, pembrolizumab showed a dramatic long-lasting response to end-stage advanced CRPC. Taking advantage of the access to initial diagnostic PBx tissue, we examined if MSI-high status was present at an earlier time point during the clinical course. We confirmed MSI-high status as well as loss of MSH2 and MSH6 proteins in the PBx tissue, consistent with dMMR likely present as a truncal defect in this patient's prostate cancer, which is in agreement with the findings of previous studies (Guedes et al. 2017; Fraune et al. 2020). This suggests that if the patient could have been tested earlier and treated with pembrolizumab, many invasive procedures, such as cystostomy, transverse colon conduit surgery, cardiac valve replacement, and colostomy surgery, could have been avoided. Currently, multiple clinical trials are ongoing to test the combination of effective treatments in CRPC for castration-sensitive PC, including ARATs, poly-ADP ribose polymerase inhibitors, CPIs, and lutetium 177-PSMA. Considering the severe clinical course before pembrolizumab administration and the marked effect of pembrolizumab in the present case, upfront usage of pembrolizumab might be a good option for some patients with advanced metastatic or unresectable PC. Further accumulation of evidence and development of optimal biomarkers are necessary to determine the optimal timing to start pembrolizumab for patients with MSI-high PC.

In the present study, following one course of pembrolizumab, the patient developed a fistula between the PC-invaded rectum and his left buttock; however, the fistula disappeared quickly after colostomy surgery. Considering the dramatic decrease of PSA level and the rapid radiographic shrinkage of the tumor after pembrolizumab treatment, the tumor infiltrating the perirectal tissue likely regressed abruptly, leaving a fistula through which abscess was drained. Thus, we speculate that the fistula may have developed because of the acute effect of pembrolizumab rather than tumor progression.

In conclusion, the present case highlights the clinical effectiveness of pembrolizumab for advanced MSI-high PC even after acquiring resistance to the last line cabazitaxel treatment. Because a dramatic and long-term effect can be observed in some patients, MSI status should actively be evaluated in all CRPC patients including those who failed other treatments. Further studies are needed to determine the optimal patient selection criteria and the timing of administration of pembrolizumab in MSI-high PC.

## METHODS

### DNA Extraction

Genomic DNA was extracted after manual microdissection using the DNA Blood and Tissue Kit (QIAGEN) according to the manufacturer's recommendations. DNA content was measured using a NanoDrop spectrophotometer (Thermo Fisher Scientific).

### Microsatellite Instability Tests

MSI typing was performed using the Bethesda marker panel (Boland et al. 1998) and CAT25, as previously described (Findeisen et al. 2005). High-level MSI (MSI-high) was scored if two or more markers showed MSI.

### Immunohistochemistry

Immunohistochemistry was performed on 4-µm paraffin sections. The primary antibodies specific for dMMR proteins were as follows: MSH2 (Roche G219-1129), MLH1 (Roche M1), MSH6 (Abcam EPR3945), and PMS2 (Roche A16-4). All dMMR protein staining was performed using an autoimmunostainer (BenchMark Ultra, Ventana Medical Systems).

### Analysis of Whole-Exome Sequencing Data

Whole-exome sequencing was performed using 1μg of frozen tumor tissue and matched leukocytes. DNA was sheared to a 200-bp peak target size, and the purified library was hybridized to the Agilent SureSelect Human All Exon v6 chip (Agilent Technologies). Paired-end sequencing (150 bp) was performed using NovaSeq6000 (Illumina). Mutation analysis was performed using Genomon2 (https://genomon-project.github.io/GenomonPagesR/). For candidate SNVs and insertions and deletions (indels) detection, the following criteria were applied: (1) variant allele frequency (VAF) of ≥5% after removing base calls with base quality or mapping quality of <20, (2) minimum number of variant-supporting consensus reads of 5, (3) a minimum read depth of 10, and (4) *P*-values for Fisher's exact test for assessing the difference in the VAF between the tumor and matched leukocyte samples of <0.01. In determining the pathogenicity, in addition to frameshift deletions and nonsense variants, any missense variants identified in COSMIC as recurrent variants in prostate cancer or classified as pathogenic/likely pathogenic in ClinVar were also considered deleterious. In addition, missense variants that met the following two criteria were defined as deleterious: (i) predicted to be deleterious in FATHMM and (ii) CADD Phred score of ≥15 (Mizuno et al. 2021). Copy-number alterations were analyzed from the sequencing data using the software CNVkit (v0.9.6). We used a coverage log_2_ ratio cutoff of ±0.3 to define gain/loss for a gene and a cutoff of ±0.8 to define amplification/deletion (Mizuno et al. 2021). The lists of the variants identified in frequently altered genes in PC are provided as Table 1 and Supplemental Table S1.

## ADDITIONAL INFORMATION

### Data Deposition and Access

The raw sequencing data have been deposited in the Japanese Genotype-phenotype Archive (JGA; http://trace.ddbj.nig.ac.jp/jga), which is hosted by the DNA Data Bank of Japan (DDBJ), under the accession number JGAS000510.

### Ethics Statement

The study complied with the Declaration of Helsinki and was approved by the Institutional Ethics Committee of Kyoto University Hospital, and written informed consent for publication of this work was obtained from the patient.

### Acknowledgments

We thank Editage (www.editage.com) for English language editing.

### Author Contributions

K.S., T.Sa., and S.A. were responsible for manuscript writing. S.A., K.M., and T.Su. were responsible for the collection and interpretation of the gene analysis data. S.A. obtained funding for the study. M.F. performed the pathological experiments and data analysis. S.A., K.M., T.G., A.S., K.I., O.O., and T.K. provided critical review and approval of the manuscript. All authors read and approved the final manuscript.

### Funding

This study was supported by Grants-in-Aid for Scientific Research, grant number 20H03814, from the Japan Society for the Promotion of Science.

### Competing Interest Statement

The authors have declared no competing interest.

## Supplementary Material

Supplemental Material
