# Supplementary material for: A case of microsatellite instability-high clinically advanced castration-resistant prostate cancer showing a remarkable response to pembrolizumab sustained over at least 18 months
Source: Cold Spring Harb Mol Case Stud. 2022 Jun;8(4):a006194. doi: 10.1101/mcs.a006194 (PMC9235847; doi:10.1101/mcs.a006194)
Supplement: Supplemental Material [file supp_8_4_a006194__DC1.html]

A case of microsatellite instability-high clinically advanced castration-resistant prostate cancer showing a remarkable response to pembrolizumab sustained over at least 18 months — Supplemental Material 

# A case of microsatellite instability-high clinically advanced castration-resistant prostate cancer showing a remarkable response to pembrolizumab sustained over at least 18 months

## Supplemental Material

- Supplemental\_Table\_S1.xlsx
